# Heterologous Expression of MYB Gene (*Rosea1*) or bHLH Gene (*Delila*) from *Antirrhinum* Increases the Phenolics Pools in *Salvia miltiorrhiza*

**DOI:** 10.3390/ijms252211917

**Published:** 2024-11-06

**Authors:** Qian Tian, Wei Han, Donghao Wang, Zhezhi Wang

**Affiliations:** Key Laboratory of the Ministry of Education for Medicinal Resources and Natural Pharmaceutical Chemistry, National Engineering Laboratory for Resource Development of Endangered Crude Drugs in Northwest of China, Shaanxi Normal University, Xi’an 710062, China; tianqian123@snnu.edu.cn (Q.T.);

**Keywords:** *Salvia miltiorrhiza*, *Antirrhinum Rosea1*/*Delila*, phenylpropanoid metabolites, phenolic acid, salvianolic acid B

## Abstract

Phenolic acids have health-promoting properties, however, but their low concentrations in *Salvia miltiorrhiza* limit broader medicinal applications. MYB and bHLH transcription factors activate multiple target genes involved in phenylpropanoid metabolism, thereby enhancing the production of various secondary metabolites. We introduced the MYB transcription factor *Antirrhinum Rosea1* (*AmROS1*) or *Delila* (*AmDEL*) into *S. miltiorrhiza* and observed that antioxidant activity in transgenic plants increased by 1.40 to 1.80-fold. The total content was significantly higher in transformants compared to the controls. Furthermore, heterologous expression of *AmROS1* or *AmDEL* triggered moderate accumulations of rosmarinic acid and salvianolic acid at various growth stages. Levels of total phenolics, total flavonoids, and anthocyanins were significantly elevated. These biological and phytochemical alterations were correlated with the upregulated expression of genes involved in phenolic acid biosynthesis. Our findings demonstrate that *AmROS1* and *AmDEL* function as a transcriptional activator in phenolic acids biosynthesis. This study offers further insights into the heterologous or homologous regulation of phenolics production, potentially enabling its engineering in *S. miltiorrhiza*.

## 1. Introduction

Secondary metabolites are compounds that do not directly participate in the normal growth, development, or reproduction of organisms. These compounds exhibit extreme diversity across various plant species and are widely utilized by humans for medicines, flavorings, and recreational drugs. As researchers strive to enhance the production of efficacious secondary metabolites in medicinal plants, they must understand how to augment their concentrations. Several strategies have demonstrated effectiveness in promoting the expression of transcription factors (TFs) [1]. By modulating the expression profiles of several target genes, MYB and bHLH proteins can govern secondary metabolism [2]. In *Arabidopsis*, the biosynthesis of flavonoids, anthocyanins, and proanthocyanidins follows a common upstream core phenylpropanoid pathway. Specific MYB and bHLH TFs significantly regulate the expression of key enzymatic genes within this pathway [2,3].

Rosmarinic acid (RA) is a prevalent chemical compound found in species of the Boraginaceae and Lamiaceae families. Its biosynthesis occurs through the tyrosine-derived and phenylpropanoid pathways [4]. Salvianolic acid B (Sal B), a dimer of RA, is an ingredient unique to *Salvia miltiorrhiza* [4,5]. Both components exhibit significant antioxidant properties and pharmacological activities. They are the primary phenolic acids in this well-known medicinal plant that is used for treating cardiovascular diseases and other ailments in China [4]. Inducing the heterologous expression of specific TFs, particularly MYBs and bHLHs, in *S. miltiorrhiza*, effectively promote the development of germplasms that produce high levels of RA and Sal B across various species. For example, the over-expression of AtPAP1 (production of anthocyanin pigment 1) and functionally similar genes enhances the accumulation of anthocyanin, proanthocyanidin, phenolics, and other phenylpropanoid metabolites in rose, *Morella rubra* [6,7], peach [8], and *S*. *miltiorrhiza* [5].

A common characteristic of MYB transcription factors is the presence of a conserved MYB domain at the N-terminus, composed of three repeated *α*-helices (R) that bind to target DNA. R2R3-MYB members are the most typical MYB TFs and function as transcription activators to regulate flavonoid biosynthesis in plants. AtMYB4 can inhibit the expression of the phenylalanine biosynthesis-related gene ADT6 and regulate the biosynthesis of flavonoids in *A.thaliana* [9]. Over-expression of CmMYB8 can restrain the expression of CHS, CHI, F3H, F3′H and DFR, resulting in a significant decrease in rutin, isorhamnetin, quercetin and kaempferol levels in *Chrysanthemum morifolium* [10].

Different tea varieties exhibit varying levels of gallic acid and catechin accumulation. CsMYB2 and CsMYB26 are differentially expressed and regulate the expression of F3′H and LAR genes in *Camellia sinensis*, respectively [11]. PpMYB17 binds to the promoters of functional genes (FLS, CHS, CHI and F3H), activating the transcription and increasing the content of flavonols, flavanols, flavonoids, isoflavones and anthocyanins in *Pyrus pyrifolia* × *Pyrus communis* callus [12]. MsMYB741 binds to the *cis*-elements of PAL/CHI gene promoter, activates gene expression and promotes the flavonoids accumulation in *Medicago sativa* roots [13].

Meanwhile, bHLH can significantly influence the accumulation of secondary metabolites. The bHLH TF contains a highly conserved bHLH domain of 50–60 amino acids, divided into two functional regions. The N-terminus regions consists of 10–15 basic amino acids that bind to *cis*-elements of DNA and regulate target gene expression. The helix-loop-helix (HLH) region at the C-terminus mediates the interaction of bHLH proteins to form homodimers or heterodimers. PabHLH1 activates the expression of CHI, CHS, FLS, DFR, and F3′H genes, thereby promoting the biosynthesis of flavonols and anthocyanins in *Plagiochasma appendiculatum* [14]. DcTT8 regulates anthocyanin accumulation by binding to the promoters of the F3′H and UFGT genes [15]. Following the transformation of EbbHLH80 into tobacco, the total flavonoid content was significantly up-regulated, and 98 flavonoid components exhibited differential enrichment [16]. MYB, bHLH, and WD40 often form a ternary complex (MBW) to play a regulatory role, which has become a consensus in higher plants. In rice, bHLH acts as a major gene that activates MYB expression, which in turn activates WD40. Through cooperation within the MBW complex, the expression of CHS, CHI, F3H, F3′H, and ANS is effectively activated, regulating anthocyanin accumulation [17,18]. The regulatory effects of MYB, bHLH and WD40 on flavonoid biosynthesis can manifest in various forms. These effects can occur either through direct binding to functional gene promoters or by forming MBW complexes via protein-protein interactions.

The expression of *AmROS1* or *AmDEL* is linked to the accumulation of anthocyanins and flavonoids [19,20], and independently promotes the expression of biosynthetic genes, including CHS. When expressed with *AmDEL*, the two can positively regulate the transcript levels of SlPAL (phenylalanine ammonia-lyase) and SlC3H (coumarate 3-hydroxylase), as well as enzyme activity and anthocyanin accumulation in tomatoes [20]. When co-expressed with *AmROS1* and *AmDEL* in *S. miltiorrhiza*, a significant number of detected enzyme genes exhibited higher expression levels. Additionally, the total phenolics, flavonoids, and anthocyanins increased significantly, while both RA and Sal B showed a marked improvement [21]. Could the expression of either *AmROS1* or *AmDEL* alone induce the accumulation of RA and Sal B? Therefore, we investigated the overexpression of *AmROS1* and *AmDelila* driven by the cauliflower mosaic virus 35S promoter in *S. miltiorrhiza*. We determined the downstream components of phenylpropanoid metabolites, including total phenolics (RA and Sal B), total flavonoids, and anthocyanins. RT-qPCR was employed to monitor the expression of several key structural genes in the phenylpropanoid pathway, and the accumulations of RA and Sal B were evaluated to determine the influence of *AmROS1* and *AmDelila.*

## 2. Results

### 2.1. Identification of Transgenic Salvia miltiorrhiza Plants

**Figure 1:**
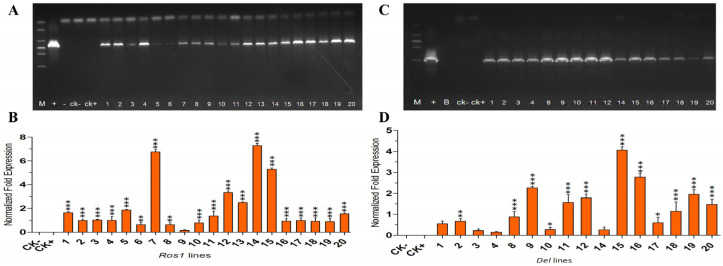
Representative PCR analyses confirmed the presence of *ROS1*, using DNA as templates. (**A**)**,** Representative PCR analyses for presence of *ROS1*, using DNA as templates. Lanes: M, DL2000 DNA Marker (100–2000 bp); 1–20, transgenic lines; +, positive control (pROS1 plasmid); −, no-template control; CK−, WT; CK+, control (pCAMBIA1302-transformed lines). (**B**), Expression of *ROS1* analyzed by RT-qPCR analysis. (**C**), Representative PCR analyses confirmed the presence of *DEL*, using DNA as templates. Lanes: M, DL200 0 DNA Marker (100–2000 bp); 1–4, 8–12, 14–20, transgenic lines; +, positive control (pDEL plasmid); −, no-template control; CK−, WT; CK+, control (pCAMBIA1302-transformed lines). (**D**), Expression of *DEL* analyzed by RT-qPCR analysis. *, ** and *** indicate that the difference is significant at *p* < 0.05, *p* < 0.01 and *p* < 0.001, respectively, between controls and transgenics.

Using PCR, we confirmed the successful generation of 20 and 16 independently derived T0 hygromycin B-resistant *Salvia miltiorrhiza* plants (Figure 1A,C). Reverse-transcription PCR indicated that varying expression levels of ROS1 and DEL across transgenic plants. RT-qPCR demonstrated that significant differences in relative quantitative expression levels among the transgenic plantlets and the controls, with the highest transcript levels found in lines ROS-7, ROS-14 and ROS-15, DEL-9, DEL-15 and DEL-16 (Figure 1B,D). None of the plants exhibited morphological changes, indicating that the gene did not affect normal growth and development but instead altered phytochemical distributions in *S. miltiorrhiza*.

### 2.2. Determination of RA and Sal B Contents in Transgenic Plants by HPLC

Whole plants from both the transgenic and control groups were dried and ground into a fine powder. These samples were repeatedly extracted and analyzed using HPLC. After 60 days of growth on the MS medium, rosmarinic acid played a dominant role in the development of plants from all lines, while levels of salvianolic acid B were lower. For instance, the RA and Sal B contents in ROS1-14 were 113.28 ± 2.20 mg ^−1^ and 11.72 ± 0.93 mg ^−1^,respectively, while DEL-15 had 76.16 ± 0.84 mg ^−1^ RA and 9.18 ± 0.18 mg ^−1^ Sal B, compared to 63.35 ± 1.90 mg ^−1^ RA and 5.86 ± 0.10 mg ^−1^ Sal B in the CK− control (Table 1). The level of RA was significantly higher in all transgenic lines compared to the controls, with the overall impact of *AmROS1* being more significant than that of *AmDEL*. For example, RA accumulated to 95.38 ± 3.24 mg g^−1^ and 94.50 ± 3.44 mg g^−1^ in lines ROS1-7 and ROS1-15, representing at least a 1.49-fold increase. While the three DEL lines showed only a 1.14- to 1.20-fold increase compared to the controls (Table 1). The Sal B content in Line ROS1-7 was 11.77 ± 1.24 mg g^−1^, reflecting a 2.0-fold increase, while the maximum value among the three DEL lines was 9.18 ± 0.18 mg g^−1^ at Day 60 (Table 1).

### 2.3. Contents of Total Phenolics, Flavonoids, and Anthocyanin, and Antioxidant Activities

**Figure 2:**
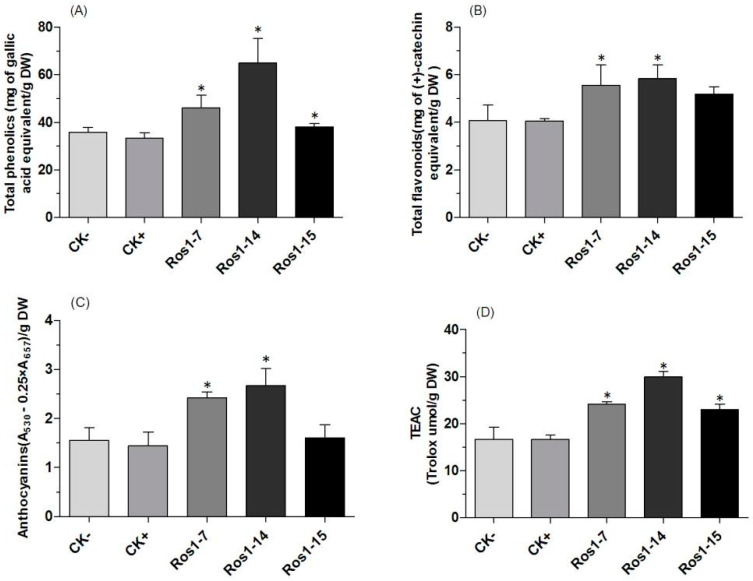
Contents of total phenolic (**A**), total flavonoid (**B**), and anthocyanin (**C**), and trolox equivalent antioxidant capacity (**D**) in extracts from CK−, WT; CK+, control; and transgenic lines ROS1-7, ROS1-14, and ROS1-15. * indicates significant difference at *p* < 0.05 between controls and transgenics.

**Figure 3:**
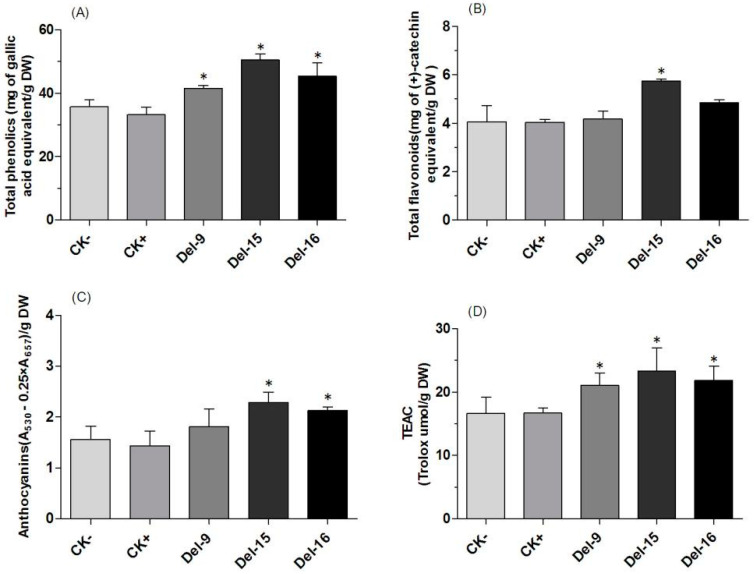
Contents of total phenolic (**A**), total flavonoid (**B**), and anthocyanin (**C**), and trolox equivalent antioxidant capacity (**D**) in extracts from CK−, WT; CK+, control; and transgenic lines DEL-9, DEL-15, and DEL-16. * indicates significant difference at *p* < 0.05 between controls and transgenics.

RA and Sal B, the active ingredients in *S. miltiorrhiza*, are derived from phenylpropanoid metabolism, as are other phenolic compounds. The total phenolics contents were significantly enhanced in transgenic lines compared to the controls, with the best performance (1.8-fold and 1.41-fold increases) observed in Lines ROS1-14 and DEL-15 (Figure 2A and Figure 3A). Total flavonoid levels were higher in all transgenic lines, although the increase was not significant in ROS1-15 and DEL-9/16 compared to the controls. The largest improvement, 1.44-fold and 1.41-fold, was recorded in Lines ROS1-14 and DEL-15, respectively (Figure 2B and Figure 3B). Anthocyanins play important roles in plant growth and development. The content did not show significant improvement in Line ROS1-15 and DEL-9, while ROS1-14 and DEL-15 exhibited only a 1.71- and 1.47-fold increase over the controls (Figure 2C and Figure 3C). The increased levels of these compounds were correlated with certain physiological indexes. For example, antioxidant activities were higher in all three transgenic lines compared to the controls, with ROS1-14 and DEL-15 demonstrating a 1.80- and 1.40-fold increase, respectively (Figure 2D and Figure 3D). The antioxidant activity results across different lines are consistent with the trend of increasing phenolic acid content.

### 2.4. Key Genes for Flavonoid Synthesis and Expression of Related Genes

**Figure 4:**
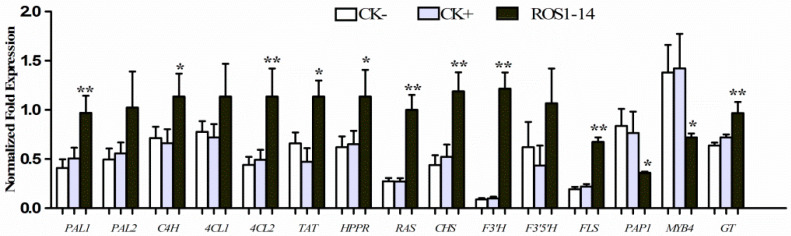
Fold-changes in expression of key structural genes for flavonoid synthesis and related functions normalized in transgenic plants. *PAL*, phenylalanine ammonia-lyase; *C4H*, cinnamate 4-hydroxylase; *4CL*, 4-coumarate-CoA ligase; *TAT*, tyrosine aminotransferase; *HPPR*, hydroxyphenylpyruvate reductase; *RAS*, rosmarinic acid synthase; *CHS*, chalcone synthase; *F3′H*, flavonoid 3′-hydroxylase; *F3′5′H*, flavonoid 3′ 5′-hydroxylase; *FLS*, flavonol synthase. * and ** indicates significant difference at *p* < 0.05 and *p* < 0.01, respectively, between controls and ROS1-14.

Utilizing Line ROS-14 and DEL-15, we evaluated the levels of RA, Sal B, total phenolics, flavonoids, and anthocyanins, while quantifying the expression of key structural genes in the phenylpropanoid pathway. All transcripts of the structural genes examined in the upstream core phenylpropanoid pathway and the upstream tyrosine-derived pathway were upregulated (Figure 4). For instance, the mRNA transcription of SmPAL1, Sm4CL2 (4-coumarate-CoA ligase), and SmRAS (rosmarinic acid synthase) was strongly induced by the heterologous expression of *AmROS1* and *AmDEL*, and SmC4H (cinnamate 4-hydroxylase), SmTAT (tyrosine aminotransferase), and SmHPPR (hydroxyphenylpyruvate reductase) were also significantly expressed. Although the expression of SmPAL2 and Sm4CL1 exhibited an increasing trend, there were no significant differences between the transgenic lines and the controls. In contrast, other genes associated with flavonoid biosynthesis, such as SmCHS (chalcone synthase), SmF3′H (flavonoid 3′-hydroxylase), SmFLS (flavonol synthase) showed a more pronounced enhancement.

## 3. Discussion

Phenolic acids derived from *Salvia miltiorrhiza* are widely used for clinical treatments. Zhang et al. [5] and Gao et al. [16] have reported that expression of AtPAP1 and bHLH can markedly enhance the production of phenolic acids in that species. However, TFs might not exhibit similar activity or could retain only a singular function in other plants [5,7,22]. Therefore, it is essential to investigate the transformation of other MYB/bHLH-regulatory genes into *S. miltiorrhiza* to determine if the phenolic acid content can be further enhanced. Upon introducing *AmROS1* or *AmDEL* into *S. miltiorrhiza*, the results were immediately apparent. Specifically, the levels of RA and Sal B increased approximately 2-fold and 1.5-fold, respectively, in the transgenic plants at both the seedling and mature stages (Table 1), Moreover, *AmROS1* exhibited a more pronounced effect compared to *AmDEL*, suggesting that MYB may play a more significant role in the accumulation of secondary metabolism in *S. miltiorrhiza*. Although *AmROS1* and *AmDEL* are known to regulate anthocyanin biosynthesis, the modest increase in anthocyanin levels (1.47-fold in DEL-15) suggests that *AmDEL* might be involved in a more complex regulatory mechanism in transgenic plantlets. *AmROS1* and *AmDEL* could interact with other endogenous regulatory elements or modulate the activity of similar transcription factors to promote the accumulation of secondary metabolites, Alternatively, they may trigger post-transcriptional modifications and or alter the expression of certain proteins through less well-understood mechanisms. Meanwhile, in comparison to previous research [19,20,21], the expression of a single gene (either *AmROS1* or *AmDEL*) resulted in a lower accumulation of Sal B than the combined expression of both genes. Although we examined only the T_0_ generation, no apparent phenotypic differences were observed between the control and transgenic plants. For both control and transgenic plants, RA can be considered a precursor of Sal B, Therefore, the results will determine whether RA is being synthesized or accumulated. In this study, RA was the dominant compound in 60-day-old seedlings that were cultured on a simple medium. However, Danshen accumulates higher levels of salvianolic acid in the natural environment, where environmental conditions are more complex, including uneven illumination and improper watering. In such conditions, the seedlings had to adapt to various stresses, necessitating the production of more secondary metabolites. Thus, levels of Sal B and several other components sharply increase, while their RA contents were either maintained at a constant level or else increased only slightly in some lines. Therefore, we can conclude that the expression of *AmROS1* and *AmDEL* activates the phenylpropanoid metabolic pathway in *S. miltiorrhiza*, playing a crucial role in the accumulation of phenolic acids. In addition, the patterns of Sal B accumulation, as revealed in our controls and transformed plants, are helpful for studying the downstream key genes that control Sal B synthesis.

We also noted that some of the key enzymatic genes in the phenylpropanoid metabolic pathway were up-regulated, either directly or indirectly, particularly SmPAL1, Sm4CL2, and SmRAS. As the primary enzyme for RA synthesis from branch pathways to the overall pathway, the observed increase in SmRAS expression aligns with previous reports on AtPAP1 [5]. This indicates that further research into its gene is warranted to understand its role in Sal B synthesis. We also found that up-regulation of the downstream critical genes for flavonoid synthesis was more obviously stimulated by *AmROS1*.

We further confirmed that the heterologous expression of the MYB/bHLH-related TF, either individually or together, can enhance the composition of secondary metabolites in *S. miltiorrhiza*. Therefore, to further enhance the content of phenolic acids, we recommend conducting additional experiments with other MYB TFs and their interaction proteins bHLH and WD40 [17,18]. In some Chinese herbal medicines, the most effective medicinal ingredients are specific metabolites, anthocyanin, and other flavonoids derived from core phenylpropanoid metabolism. Therefore, additional TFs might serve as valuable tools for investigating the effects of heterologous expression.

## 4. Conclusions

The MYB transcription factor *Antirrhinum Rosea1* (*AmROS1*) or *Delila* (*AmDEL*) have been shown to enhance the accumulation of RA and Sal B in *S. miltiorrhiza*. Notably, *AmROS1* exhibits a stronger effect on promoting the biosynthesis of phenolic acid metabolites and the accumulation of other flavonoids and anthocyanins compared to *AmDEL*. Gene expression analysis in transgenic plants suggests that RAS might play a crucial role in regulating phenolic acid biosynthesis. Further investigation into other MYB/bHLH transcription factors or related regulatory proteins could provide insights into secondary metabolite production in *S. miltiorrhiza*.

## 5. Materials and Methods

### 5.1. Plant Material, Vector Construction, and Transformation in Salvia miltiorrhiza

The *Salvia miltiorrhiza* Bunge seeds were collected from Tianshili Danshen Medicinal Source Base in Shangluo County, Shaanxi Province, China. The mature seeds of *S. miltiorrhiza* were surface-sterilized using 0.1% mercuric chloride (HgCl_2_) and subsequently germinated on a 1/2-strength MS basal medium (25 ± 2 °C, 16-h photoperiod, 25 μmol m^−2^ s^−1^). One-month-old seedlings were utilized for plant transformation experiments. The genes *AmROS1* (DQ275529) and *AmDEL* (M84913) were cloned from the cDNA of *Antirrhinum majus* flowers with primers ROS1F/ROS1R and DEL1F/DEL1R (Supplementary Table S1). Following the introduction of the pMD19-T-vector (TaKaRa, Dalian, China) into *E. coli DH5a*, sequencing was performed for verification. The plasmids pROS and pDEL were ligated with the fragments of pCAMBIA1302 digested with Bgl II and BstE II, using T4 DNA ligase. The expression vectors pROS1 and pDEL (pCAMBIA1302-ROS1, pCAMBIA1302-DEL) were constructed for using in *Agrobacterium* strain EHA105-mediated transformation of *S. miltiorrhiza* established in our laboratory [23].

### 5.2. Identification of Transgenic Plants via PCR and RT-Q-PCR

When the transgenic and wild plants reached the four true leaf stage in our tissue culture room, one leaf was collected for DNA analysis, and the entire plantlet was harvested for RNA extraction. Genomic DNA and RNA were isolated using E.Z.N.A.^TM^ according to the manufacturer’s instructions. First-strand cDNA was synthesized using a PrimeScript^®^ RT reagent Kit (TaKaRa). The presence of transgenic plants was confirmed by PCR amplification, using genomic DNA/RNA as templates and gene-specific primers ROS1F/ROS1R, DEL1F/DEL1R, and GFPF/GFPR. The primers used for all procedures are listed in Supplementary Table S1. Real-time quantitative PCR (RT-qPCR) was performed using an iQ5 Thermocycler (Bio-Rad, Hercules, CA, USA) under the following conditions: an initial pre-denaturation at 95 °C for 1 min, followed by 40 cycles of denaturation at 95 °C for 10 s and annealing at 60 °C for 30 s. The ACTIN (DQ243702) served as a control and was amplified with primers SmACTF/SmACTR. Gene expression levels were quantified using the comparative C_T_ method [24]. Each PCR reaction was performed four times using independent samples.

### 5.3. Extraction and HPLC Analysis of Phenolic Compounds, Determination of Total Phenolics, Total Flavonoids, and Anthocyanins, and Monitoring of Antioxidant Activities

Plant roots materials were collected after being grown for 60 days on an MS basic medium at 25 ± 2 °C. The wild type (WT, CK−), control (pCAMBIA1302, CK+), and three *AmROS1* transgenic lines (ROS-7, ROS-14 and ROS-15) as well as three *AmDEL* transgenic lines (DEL-9, DEL-15 and DEL-16) were analyzed by HPLC. We measured the contents of total phenolics, total flavonoids, and anthocyanins, and assessed antioxidant activities. The methodology for determining total phenolics and flavonoids has been previously detailed [25]. Anthocyanin quantification followed the protocol by Mano et al. [26]. Antioxidant activities for methanol extracts from both transgenic and wild-type roots were evaluated using the trolox equivalent antioxidant capacity (TEAC) assay. This assay evaluates the ability of antioxidant molecules to quench the long-lived ABTS (2,20-azinobis 3-ethylbenzthiazoline-6-sulfonate) radical cation, a blue-green chromophore with a characteristic absorption at 734 nm. Results was compared to those for trolox (6-hydroxy-2,5,7,8-tetra-methylchroman-2-carboxylic acid), a water-soluble vitamin E analogue. Antioxidant capacity was expressed as TEAC, measured in millimoles of trolox per gram of dry weight. All collected samples were treated according to the protocol of Zhang et al. [5]. Five independent samples were processed separately. One-way analysis of variance (ANOVA) followed by Tukey’s multiple comparisons test was conducted using SPSS23 with differences between means considered significant at the 5% confidence level.

## Figures and Tables

**Table 1:** Variations in contents of rosmarinic acid (RA) and salvianolic acid B (Sal B) from transgenic lines (ROS1-7, ROS1-14, ROS1-15 and DEL-9, DEL-15, DEL-16) and controls (CK−, WT; CK+, control from pCAMBIA1302 transformed lines) at different growth stages. *, ** and *** indicate that the difference is significant at *p* < 0.05, *p* < 0.01 and *p* < 0.001, between controls and transgenics.

Compound	CK−	CK+	ROS1-7	ROS1-14	ROS1-15	DEL-9	DEL-15	DEL-16
RA	63.35 ± 1.90 mg g^−1^	62.15 ± 2.51 mg g^−1^	95.38 ± 3.24 mg g^−1^ ***	113.28 ± 2.20 mg g^−1^ ***	94.50 ± 3.44 mg g^−1^ ***	72.21 ± 0.28 mg g^−1^ **	76.16 ± 0.84 mg g^−1^ ***	72.82 ± 0.41 mg g^−1^ **
Sal B	5.86 ± 0.10 mg g^−1^	5.71 ± 0.50 mg g^−1^	11.77 ± 1.24 mg g^−1^ ***	11.72 ± 0.93 mg g^−1^ ***	5.24 ± 0.61 mg g^−1^	8.55 ± 0.40 mg g^−1^ *	9.18 ± 0.18 mg g^−1^ **	8.54 ± 0.78 mg g^−1^ *

## Data Availability

Data is contained within the article or Supplementary Materials.

## Supplementary Materials

The following supporting information can be downloaded at: https://www.mdpi.com/article/10.3390/ijms252211917/s1.
