# Supplementary material for: Heterologous Expression of MYB Gene (Rosea1) or bHLH Gene (Delila) from Antirrhinum Increases the Phenolics Pools in Salvia miltiorrhiza
Source: Int J Mol Sci. 2024 Nov 6;25(22):11917. doi: 10.3390/ijms252211917 (PMC11593512; doi:10.3390/ijms252211917)
Supplement: Supplementary file 1 [file ijms-25-11917-s001.zip › ijms-3262705-supplementary.pdf]

Supplementary Table S1. Primers for sequences

	Direction	Sequence (5'→3')
Detected primer	ROS1F	5'-GAAGATCTATGGAAAAGAATTGTCGTGG AGT-3' (*Bgl* II underlined)
	ROS1R	5'-CAGGGTAACCTTAATTTCCAATTTGTTGG GCCT-3' (*Bst*E II underlined)
	DEL1F	5'-GAAGATCTGGATCCATGGCTACTGGTATC CAAAACCAAAAG-3'(*Bgl* II and *Bam*H I underlined)
	DEL1R	5'-CAGGGTGACCGAGCTCAACTTCAAGACTT CATAGTAACTTTCTG-3'(*Bst*E and II *Sac* I underlined)
	GFPF	5'-AAGGAGAAGAACTTTTCACTGGAG-3'
	GFPR	5'-AGATTGTGTGGACAGGTAATGGTT-3'
RT-Q-PCR primer	RT-ROS1F	5'-AAATGGTCGCTGATTGCTGGTA-3'
	RT- ROS1R	5'- CGTTCTCCATCCTCGCCTAAAT-3'
	RT-DEL1F	5'- CAGTATGGCTATGCAACGCTCA-3'
	RT- DEL1R	5'- CTATCAATTTCATCAGCCGTTGG-3'
	SmACTF	5'- AGGAACCACCGATCCAGACA-3'
	SmACTR	5'- GGTGCCCTGAGGTCCTGTT-3'
	SmPAL1-F	5'-GATAGCGGAGTGCAGGTCGTAC-3'
	SmPAL1-R	5'-CGAACTAGCAGATTGGCAGAGG-3'

	SmPAL2-F	5’-GGCGGCGATTGAGAGCAGGA-3’
	SmPAL2-R	5’-ATCAGCAGATAGGAAGAGGAGCACC-3’
	SmC4H-F	5’-CCAGGAGTCCAAATAA CAGAGCCG-3’
	SmC4H-R	5’-GCCACCAAGCGTTCACCAAG AT-3’
	Sm4CL1-F	5’-ATTCGCATTCGCATTTCTCGG-3’
	Sm4CL1-R	5’- GCGGCGTAGTGCTTCACCTTT-3’
	Sm4CL2-F	5’- TCGCCAAATACGACCTTTCC-3’
	Sm4CL2-R	5’-TGCTTCAGTCATCCCATACCC-3’
	SmTAT-F	5’-CAACTGCTGGTCTTCCACAAAC-3’
	SmTAT-R	5’-GCGAGCCAAAACGGACA-3’
	SmHPPR-F	5’-TGACTCCAGAAACAACCCACATT-3’
	SmHPPR-R	5’-CCCAGACGACCCTCCACAAG-3’
	SmRAS-F	5’- GCAAACGAGCACCACCTATCC -3’
	SmRAS-R	5’- GTCTTGGAGCGGGGTTTCG -3’
	SmCHS-F	5’-CGCGATTATGCTTGAGGTTGA-3’
	SmCHS-R	5’-CACTACTTGATGTCCCATTTCTTGAC-3’
	SmF3’H-F	5’-TCAGGCTTTGAGCAATGGGAAGT-3’
	SmF3’H-R	5’-TGAGTCGCATGGGCAGAGGAA-3’
	SmF3’5’H-F	5’-CATCTACTCCAACATCGGACAGC-3’
	SmF3’5’H-R	5’-CCCCACATAAGGTTCATCAACAG-3’
	SmFLS-F	5’-GTTCGTGCATCCCGAGTTCAA-3’
	SmFLS-R	5’-CTTCTCGTCACCTCTCGACATCTTT-3’

C4H, cinnamate 4-hydroxylase; *CHS*, chalcone synthase; *4CL*, 4-coumarate-CoA ligase; *F3'H*, flavonoid 3'-hydroxylase; *F3'5'H*, flavonoid 3',5'-hydroxylase; *FLS*, flavonol synthase; *HPPR*, hydroxyphenylpyruvate reductase; *PAL*, phenylalanine ammonia lyase; *RAS*, rosmarinic acid synthase; *TAT*, tyrosine aminotransferase.

Supplementary Figure.S1

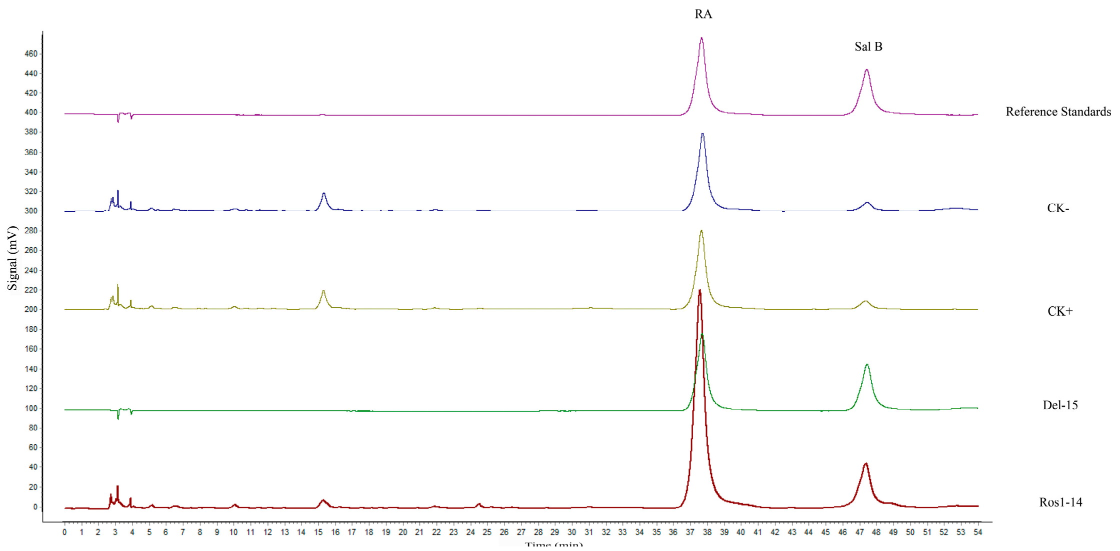

Supplementary Fig.S1. Representative HPLC chromatograms of reference Standards and 60-day-old plantlets of DEL-15,ROS-14, and control line(CK-, CK+).
